# Copper Deficiency Mimicking Myelodysplastic Syndrome: A Case Report

**DOI:** 10.7759/cureus.66765

**Published:** 2024-08-13

**Authors:** Gurjot Singh, Kanishka Goswami, Amna Farooq, Shubam Trehan, Rajpreet S Arora, Gaurav Bector, Syeda Ashna Fatima Kamal, Waqas Azhar

**Affiliations:** 1 Internal Medicine, Maharaj Sawan Singh Charitable Hospital, Beas, IND; 2 Hematology and Oncology, Southern Illinois University School of Medicine, Springfield, USA; 3 Hematology and Oncology, Memorial Medical Center, Springfield, USA; 4 Hematology and Oncology, Hospital Sisters Health System (HSHS) St. John’s Hospital, Springfield, USA

**Keywords:** copper deficiency, copper myeloneuropathy, myeloneuropathy, sideroblastic anemia, myelodysplastic syndrome

## Abstract

Copper deficiency can mimic myelodysplastic syndrome, a group of heterogeneous hematopoietic disorders characterized by peripheral cytopenias, with potential progression to bone marrow failure and acute myeloid leukemia. We present the case of an 83-year-old female with a history of Graves’ disease, early-stage hormone receptor-positive breast cancer, hypertension, and glaucoma, who presented with fatigue and progressive lower extremity weakness. Laboratory tests revealed macrocytic anemia, neutropenia, and lymphopenia, with normal platelet counts. Bone marrow biopsy showed trilineage hematopoiesis, dyserythropoiesis, ring sideroblasts, and vacuoles in erythroid precursors, indicating copper deficiency. The patient had been using zinc oxide paste for dentures and had increased her zinc intake during the COVID-19 pandemic, leading to severe copper deficiency. Treatment with intravenous and oral copper supplementation resulted in marked improvement in hematologic indices and symptoms. This case underscores the importance of considering copper deficiency in the differential diagnosis of cytopenias and myeloneuropathy in elderly patients, particularly those with a history of excessive zinc intake.

## Introduction

Myelodysplastic syndrome (MDS) represents a group of clonal hematopoietic disorders characterized by ineffective hematopoiesis, leading to peripheral blood cytopenias and a variable risk of progression to acute myeloid leukemia [1]. This syndrome primarily affects older adults, with a median age of diagnosis around 71 years, and is associated with a range of genetic and environmental risk factors, including exposure to radiation, chemotherapy, and certain chemicals [1,2]. The pathogenesis of MDS involves the clonal expansion of abnormal hematopoietic stem cells, which produce dysfunctional blood cells and impair normal bone marrow function.

Diagnosis of MDS is often complex and requires careful evaluation of clinical, laboratory, and cytogenetic data. The presence of unexplained cytopenias, dysplastic changes in the bone marrow, and characteristic genetic mutations are crucial in confirming the diagnosis [2]. However, several conditions can mimic MDS, posing a diagnostic challenge. These include reversible causes such as vitamin deficiencies (e.g., vitamin B12 and folate), medications, alcohol abuse, and infections. Among these, copper deficiency is a rare but notable cause, especially because it can present with hematologic abnormalities that closely resemble those seen in MDS [2].

Copper is an essential trace element involved in various physiological processes, including iron metabolism, neurological function, and immune response [3]. It acts as a cofactor for enzymes such as ceruloplasmin, which is critical for iron mobilization from tissues to plasma, and cytochrome c oxidase, involved in cellular respiration. Copper deficiency can lead to anemia, neutropenia, and neurological symptoms, including myeloneuropathy, characterized by progressive weakness, numbness, and sensory ataxia [3].

The absorption and metabolism of copper can be disrupted by several factors, most notably excessive intake of zinc. Zinc and copper share similar absorption pathways in the gastrointestinal tract, and high levels of zinc can inhibit copper absorption by inducing the synthesis of metallothionein, a protein that preferentially binds copper [4]. This mechanism can lead to copper deficiency in patients with high zinc intake, either from dietary supplements or medical treatments, such as zinc-containing denture creams.

In this case report, we describe an elderly woman who presented with symptoms suggestive of MDS but was ultimately diagnosed with copper deficiency induced by excessive zinc intake. This case underscores the importance of considering nutritional and metabolic causes in the differential diagnosis of hematologic abnormalities, particularly in the elderly population. Early recognition and treatment of copper deficiency can prevent unnecessary diagnostic procedures and improve patient outcomes.

## Case presentation

An 83-year-old female with a past medical history of Graves’ disease, stage 1 hormone receptor-positive breast cancer treated with lumpectomy, adjuvant radiation, and endocrine therapy, hypertension, and glaucoma presented with a complaint of fatigue and progressive weakness in the lower extremities over several months. She also reported generalized weakness and tingling in the lower extremities. Physical examination was notable for scattered ecchymoses, predominantly on the left forearm.

Laboratory findings revealed macrocytic anemia (hemoglobin: 7.0 g/dL), neutropenia (absolute neutrophil count: 450/µL), and lymphopenia (absolute lymphocyte count: 400/µL), with a normal platelet count. Additional workup showed low iron levels, elevated ferritin, and normal vitamin B12 and folate levels. The patient’s disseminated intravascular coagulation panel, along with liver and kidney function tests, were all within normal limits (Table 1). Hepatitis and HIV screenings were negative. A CT scan of the chest, abdomen, and pelvis showed normal results.

**Table 1:** Laboratory reports.

Parameters	Results	Reference range
White blood cell count (1,000/mm^3^)	1.1	4–11
Platelet count (1,000/mm^3^)	191	150–450
Red blood cell count (million/µL)	2.89	4.0–5.1
Hemoglobin (g/dL)	7.5	12–14
Mean corpuscular volume (fL)	107.5	80–100
Mean corpuscular hemoglobin (pg)	37.5	27.5–33.2
Mean corpuscular hemoglobin concentration (g/dL)	36.4	33.4–35.5
Absolute neutrophil count (1,000/mm^3^)	450	2,500–7,000
Absolute lymphocyte count (1,000/mm^3^)	400	1,000–5,000
Serum iron	20	20–40
Ferritin levels (ng/mL)	07	30–400
Vitamin B12 levels (pg/mL)	550	160–950
Reticulocyte count	0.9 %	0.5-2.5%

**Figure 1:**
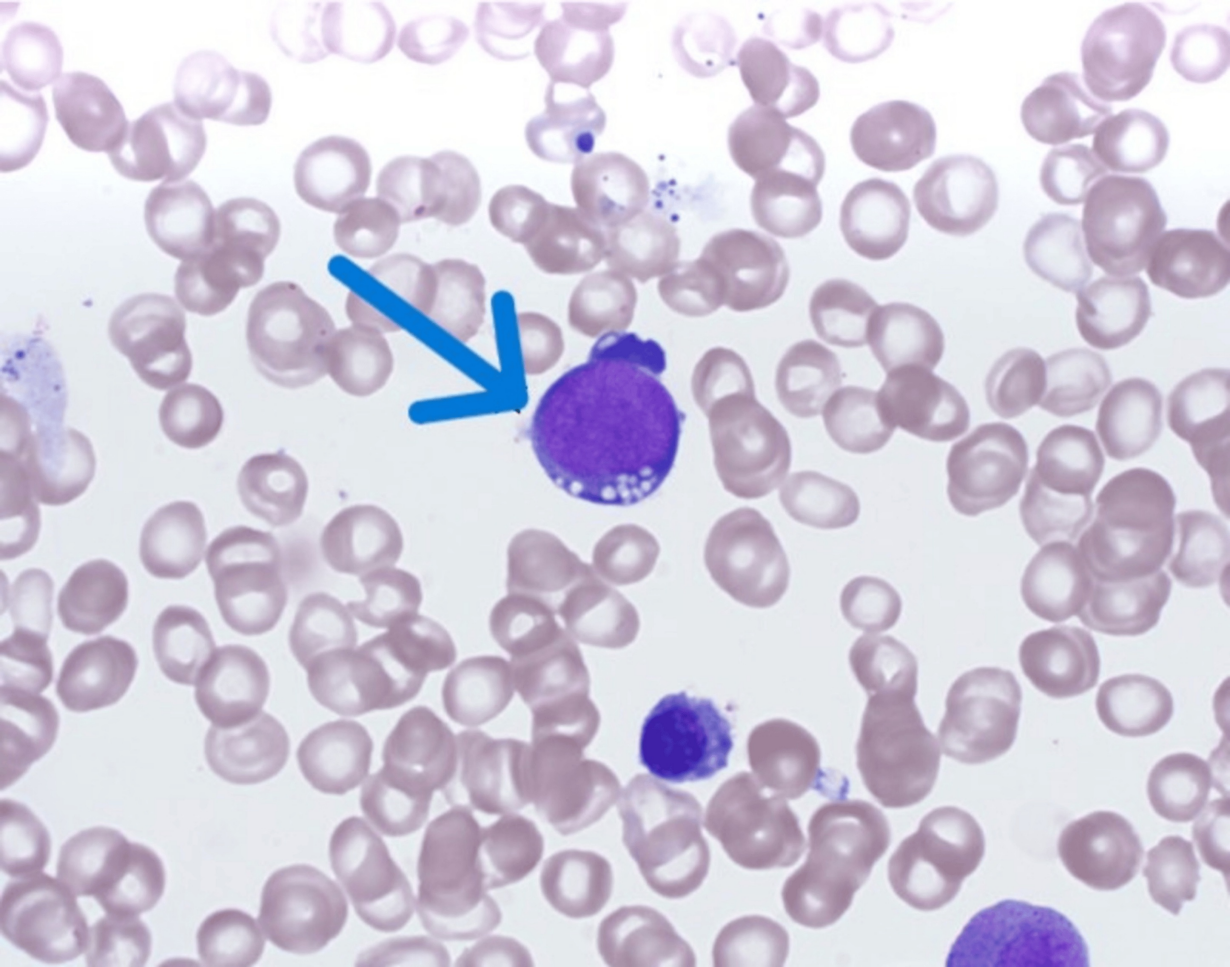
Bone marrow biopsy reveals mildly hypercellular marrow with trilineage hematopoiesis, increased erythropoiesis, mild dyserythropoietic changes, decreased storage, increased sideroblastic iron, and rare ringed sideroblasts. Vacuoles within erythroid precursors are present (arrow).

A peripheral blood smear confirmed severe anemia with mild macrocytosis, absolute neutropenia, and absolute lymphopenia, with no abnormal blast cells. Immunophenotyping ruled out B or T-cell abnormalities, excluding a clonal hematologic process. Bone marrow biopsy demonstrated mildly hypercellular marrow with trilineage hematopoiesis, increased erythropoiesis, mild dyserythropoietic changes, decreased storage iron, and increased sideroblastic iron with ringed sideroblasts (Figure 1). The presence of vacuoles in erythroid precursors suggested copper deficiency.

Further history revealed that the patient had been using zinc oxide paste for her dentures and had increased her zinc intake with supplements during the COVID-19 pandemic. Serum copper levels were found to be <10 µg/dL, while serum zinc levels were 147.8 µg/dL (normal range: 60-120 µg/dL) (Table 2).

**Table 2:** Improvement in hematologic indices with rising serum copper levels.

Parameter	Presentation	Day 1 of copper supplementation	Day 7 of copper supplementation	Day 30 of copper supplementation	Reference range
Hemoglobin (g/dL)	7.0	7.2	9.1	11.7	13.8–17.2
Mean corpuscular volume (fL)	107.5	105.0	95.0	92.0	80–100
White blood cells (×10^9^/L)	1.1	1.32	2.9	6.6	4.5–11.0
Absolute neutrophil count (×10^9^/L)	0.45	0.5	0.8	4.8	2.0–7.5
Platelets (×10^9^/L)	191	197	251	271	150–450
Copper (µg/dL)	<10	-	-	68	70–140
Zinc (µg/dL)	147.8	-	-	-	70–120

The patient was diagnosed with severe copper deficiency and was treated with intravenous copper (2 mg daily for three days), followed by oral copper supplementation. This intervention led to significant improvement in hematologic parameters. The patient also received weekly intramuscular vitamin B12 and daily oral folic acid supplements.

## Discussion

MDS predominantly affects the elderly, with a median age of diagnosis around 71 years. It results from ineffective hematopoiesis due to genetic damage in hematopoietic stem cells, potentially induced by radiation, chemotherapy, infections, or inherited genetic mutations [1,2]. In this case, the patient’s primary risk factor for MDS was age. However, several reversible conditions, including copper deficiency, can mimic MDS [2].

Copper deficiency is associated with chronic malnutrition, malabsorptive enteropathies (e.g., celiac disease), gastric and bariatric surgery, inadequate parenteral nutrition, chronic use of proton pump inhibitors, copper chelators (e.g., penicillamine), and increased zinc intake [3]. Copper and zinc compete for absorption in the intestines via intracellular ligands such as metallothionein. Although copper has a stronger affinity for metallothionein, excessive zinc intake can inhibit copper absorption, leading to deficiency [3].

Copper plays a crucial role in various physiological processes, including iron absorption and metabolism, facilitated by proteins such as hephaestin and ceruloplasmin ferroxidase. Copper deficiency impairs mitochondrial cytochrome oxidase activity, hindering heme synthesis and resulting in the formation of ring sideroblasts, alongside symptoms such as hypothermia and microcytosis [4,5]. Neutropenia in copper deficiency may result from decreased neutrophil survival or inhibited differentiation of CD34(+) hematopoietic progenitor cells [6].

Clinically, copper deficiency can present with symptoms such as muscle weakness, fatigue, neuropathy, and progressive cytopenias, distinct from MDS. Bone marrow findings may include dysplastic erythroid precursors, but the presence of cytoplasmic vacuoles in erythroid and myeloid precursors is pathognomonic for copper deficiency. Importantly, isolated copper deficiency does not exhibit the cytogenetic abnormalities typical of MDS [7].

In this case, the chronic use of zinc oxide paste and over-the-counter zinc supplements led to severe copper deficiency [8]. Recognizing this condition requires a high index of suspicion, particularly in elderly patients presenting with cytopenias and neurological symptoms [9]. Prompt treatment with copper supplementation can rapidly improve hematologic abnormalities and resolve neuropathic symptoms. Oral copper supplementation, by saturating intestinal transport systems, can effectively restore serum copper levels [10].

## Conclusions

Copper deficiency should be considered in elderly patients with unexplained cytopenias, myelodysplastic changes, ring sideroblasts, and associated myeloneuropathy. The presence of cytoplasmic vacuoles in erythroid or myeloid precursors is indicative of copper deficiency. Timely diagnosis and treatment with intravenous and oral copper supplementation can lead to the resolution of symptoms and hematologic abnormalities. It is crucial to educate patients on the risks of excessive zinc intake and the importance of discussing supplement use with healthcare providers.
